# Impacts of Self Perceived Malocclusion on the Oral Health Related Quality of Life of Young Adults

**DOI:** 10.3390/healthcare9030248

**Published:** 2021-03-01

**Authors:** Zawani Mohd Tajudin, Wan Nurazreena Wan Hassan, Zamros Yuzadi Mohd Yusof, Mohd Zambri Mohamed Makhbul

**Affiliations:** 1 Department of Paediatric Dentistry and Orthodontics, Faculty of Dentistry, University of Malaya, Kuala Lumpur 50603, Malaysia; zawani_mtajudin@siswa.um.edu.my; 2 Department of Community Oral Health and Clinical Prevention, Faculty of Dentistry, University of Malaya, Kuala Lumpur 50603, Malaysia; zamros@um.edu.my; 3 Orthodontic Unit, Klinik Pergigian Cahaya Suria, Ministry of Health Malaysia, Kuala Lumpur 50100, Malaysia; zambrimakhbul@gmail.com

**Keywords:** quality of life, ethnicity and health, gender and health, dental aesthetics

## Abstract

Self-awareness of poorly arranged teeth can influence the quality of life. This study aimed to report the impacts of self-perceived malocclusion in young adults and the association between demographic characteristics and oral health related quality of life (OHRQoL). In this cross-sectional study, six-hundred-forty-three subjects from Selangor, Malaysia selected using a multistage sampling technique answered the Psychosocial Impact of Dental Aesthetics (PIDA) questionnaire and self-rated their dental appearance using the Aesthetic Component of the Index of Orthodontic Treatment Need. Data were analyzed using multifactorial ANOVA to identify the association between demographic characteristics and total PIDA score. Five-hundred-twenty-four subjects (81.5%) completed the questionnaires. Overall, 87.8% had impacts on their OHRQoL. Psychological impact was the most impacted domain (75.8%), followed by dental self-confidence (59.4%), social impact (48.9%) and aesthetic concern (22.1%). 16.8% reported significant impacts on all domains. Their mean PIDA score was 36.3 (SD 17.1). Prevalence, extent and severity of impacts were higher amongst those with self-perceived malocclusion. Gender, ethnicity, and self-perceived malocclusion status were associated with PIDA score (*p* < 0.05). Sub-urban and rural females had significantly higher PIDA scores than sub-urban and rural males. In conclusion, majority of Malaysian young adults especially those with self-perceived malocclusion were impacted by their dental aesthetics.

## 1. Introduction

The teeth are one of the prominent structures that refines the facial aesthetics. Adults have expressed dissatisfaction with their dental appearance [1], with the younger individuals finding dental appearance more important than the elderly [2]. Malocclusion is a dental condition from poorly arranged teeth. Self-awareness of one’s malocclusion is one of the features of the teeth that has been shown to induce a negative relationship between one’s social well-being and quality of life [3,4]. Those seeking treatment perceive that orthodontics would not only straighten their teeth but also enhance their self-confidence and facial appearance through improvement of their dental health [5].

Epidemiological research to establish the prevalence of impact of malocclusion on oral health-related quality of life (OHRQoL) in populations is important to establish the perceived needs for orthodontic treatment. Past studies have established the prevalence of oral impacts associated with malocclusion in the German naval [6] and Brazilian army [7] populations while other studies have reported on the severity [8,9] and the extent [10] of the impacts of malocclusion.

When measuring the prevalence of impact of oral conditions, the use of established instruments is preferred as they will enable comparisons between different populations to be made objectively. Few instruments have been developed to assess the impact of malocclusion on the OHRQoL. Of these, the Psychosocial Impact of Dental Aesthetics (PIDA) questionnaire [11] has been translated and cross-culturally adapted into several languages. Currently, it is the only malocclusion-specific self-reported instrument that has been validated for use by Malaysians [12,13]. The use of this instrument is significant in assessing the impact of perceived malocclusion on the psychosocial well-being of individuals, which can in turn translate into motivation for seeking orthodontic treatment [13].

Past studies have looked at the prevalence of oral impact association with malocclusion amongst the orthodontic treatment seeking population [1,4,5]. However, there is a lack of prevalence studies on the impacts of malocclusion among the young adult Malaysian population, thus indicating the need for such research. Such data will be useful to determine the population-based prevalence of perceived needs related to malocclusion and for policy makers to plan for future dental workforce and financial resources. The data can also be used to compare the impacts of malocclusion with other populations.

With that in mind, the primary objective of the study was to assess the prevalence, extent, and severity of the psychosocial impact of dental aesthetics in Malaysian young adults. The secondary objectives were to compare the impacts between those with and without self-perceived malocclusion, and to investigate the association between demographic characteristics and the OHRQoL. The null hypotheses were (1) there is no significant difference in OHRQoL impacts between young adults with and without self-perceived malocclusion, and (2) there is no association between demographic characteristics and OHRQoL. The alternative hypotheses were (1) there is a significant difference in OHRQoL impacts between young adults with and without self-perceived malocclusion and (2) there is association between demographic characteristics and OHRQoL.

## 2. Materials and Methods

This was a cross-sectional study involving young adults aged 18 to 30 years in Malaysia. The participants were recruited from selected Malaysian tertiary education institutions using a multistage sampling technique. First, Malaysia was divided into five regions: northern, central, southern, east coast, and Borneo. Next, the central region was randomly selected, which consisted of three states. One state, i.e., Selangor from the central region was randomly selected. In the state of Selangor, the sampling frame consisted of students from four types of institutions: matriculations, community colleges, polytechnics, and faculties of a university. Next, one institution from each type was randomly selected. Throughout this sampling method from stage one to stage three, randomization was done using online randomizer software to eliminate potential bias [14]. Then, the final sample was randomly selected proportionate to the size of the registered students from the four selected institutions. Those who were undergoing or had had orthodontic treatment, with cleft lip and/or palate, or other craniofacial deformities, or with learning difficulties who could not read or write in English or Malay languages were excluded.

Sample size calculation was performed following several steps. First, a pretest using the PIDA questionnaire with 30 subjects from three different institutions not involved in the current study was carried out to calculate the design effect using the formula by Kerry and Bland [15]. Based on the pretest, the design effect was 3.61. Next, sample size calculation was done based on an estimated prevalence of 90.2% [10], 95% confidence interval, and 5% error giving a sample size of 136. This number was multiplied with the design effect of 3.61 and a 20% anticipated non-response rate giving the final sample size of 589 subjects.

The questionnaire used in the study comprised of three sections. Firstly, subjects reported their demographic characteristics. They then answered the Malaysian versions of the PIDA questionnaire [12,13]. This instrument consisted of 22 items measuring 4 domains: Dental Self-confidence (DSC, 6 items), Social Impact (SI, 8 items), Psychological Impact (PI, 6 items), and Aesthetic Concern (AC, 2 items). The items were scored based on a 5-point Likert scale; 0 = ‘not at all’ to 4 = ‘very strongly’. Finally, subjects self-rated their malocclusion using the Aesthetic Component of the Index of Orthodontic Treatment Need (IOTN-AC) [16], which comprised a black and white 10-point-photographic-scale from 1 as ‘very good’ to 10 as ‘very severe’.

Data collection took place between September 2017 and January 2018. The majority of the subjects answered the questionnaire in classroom settings (55.3%), while the remaining subjects answered the questionnaire outside classroom settings (27.0%) and using online questionnaires (17.7%). All study subjects had given written consent prior to the data collection.

### Data Analysis

Data analysis was done using the Social Packages for Social Sciences (SPSS) (ver. 24.0; IBM Corp, Armonk, NY, USA).

Descriptive statistics were used to describe the sample. For the PIDA questionnaire, subjects with more than 20% missing items in any domain were removed from the analysis [17]. Thus, only one missing item was allowed for the SI, DSC, and PI domains. No missing items were allowed for the AC domain. In this study, there was one item with four missing data in the DSC domain, and one item with one missing data for both the SI and PI domains.

The prevalence of PIDA was calculated as the percentage of subjects who reported a “significant impact”, i.e., a score of 3 or 4 (strong positive ratings) in any items of the SI, PI and AC domains, or a score of 0 or 1 (strong negative ratings) in any items of the DSC domain. The severity of PIDA was calculated by summing up the item scores of the SI, PI and AC domains and item reverse-scores of the DSC domain. Subsequently, mean, standard deviation, median and interquartile range were reported. The extent of PIDA was calculated as the percentage of subjects with the number of domains with at least one item having a “significant impact”. For the IOTN-AC, subjects with self-perceived malocclusion was defined as those with a score of 3 or higher in the IOTN-AC scale, while subjects without self-perceived malocclusion were those with a score of 1 or 2 in the IOTN-AC scale [16].

For the associated factors of PIDA, the demographic characteristics included in the analysis were age, gender, ethnic group, household income, and place of residence. The three main ethnic groups in Malaysia were Malay, Chinese, and Indian, along with an “Other” category for the minority ethnic groups. The household income consisted of 10 categories following the income levels described by the Department of Statistics [18]. The place of residence was classified into urban, sub-urban and rural areas.

The prevalence and extent of PIDA between subjects with and without self-perceived malocclusion were compared using the Chi-square test. The severity of PIDA were compared using independent t-test. The associated nominal or ordinal factors for PIDA were analyzed using multifactorial ANOVA to assess the relationships of demographic factors with the PIDA of Malaysian young adults. For multifactorial ANOVA analysis, the effect size of the partial eta squared was based on small (0.01), medium (0.06) and large (0.14) [19]. The relationship between age and PIDA scores was analyzed using Pearson correlation.

## 3. Results

Six hundred and eighty questionnaires were distributed but only 574 subjects responded. However, an initial check showed that 37 subjects who answered the questionnaire had orthodontic treatment and were therefore excluded from the study. As a result, the actual number of responses was 537 of the 643 distributed questionnaires (83.5% response rate). From this, 13 responses were removed from data analysis; six due to incomplete demographic data, and seven due to higher percentage of missing data from the PIDA questionnaires. Therefore, 524 subjects with complete data were included in the analysis (81.5% completion rate). Six subjects had less than 20% missing items in any of the PIDA domains. Thus, the missing scores were imputed with the mean score of the items for that particular domain [17].

### 3.1. Demographic Data

Table 1 shows the demographic characteristics of the subjects. There were more female (54.2%) than male (45.8%) subjects. The mean age was 19.49 ± 1.47 years. The majority of the subjects were Malays (88.8%), followed by Indians (5.7%), Chinese (4.0%) and others (1.5%). The distribution of household incomes were spread almost evenly, with the highest income being more than RM5000 (16.2%) and the lowest income between RM3500 to RM3999 (3.4%). The majority of the subjects lived in urban areas (57.1%), followed by sub-urban (23.3%) and rural (19.6%) areas. The majority of the subjects were without self-perceived malocclusion (58.6%) compared to subjects with self-perceived malocclusion (41.4%).

### 3.2. Prevalence of Psychosocial Impact of Dental Aesthetics among the Participants by Self-Perceived Malocclusion

Table 2 shows the prevalence of PIDA in the overall sample, and samples with and without self-perceived malocclusion. The overall prevalence of PIDA in the sample was 87.8%. The prevalence of impacts was significantly higher in subjects with self-perceived malocclusion (92.6%) than in subjects without self-perceived malocclusion (84.4%) (*p* < 0.01). Generally, the prevalence was highest in the PI domain, followed by DSC, SI and AC domains.

### 3.3. Severity of Psychosocial Impact of Dental Aesthetics among the Participants by Self-Perceived Malocclusion

Table 3 shows the mean (standard deviation) and median (interquartile range) scores of total PIDA and the respective domains for all subjects and subjects with and without self-perceived malocclusion. The mean total PIDA score for all subjects was 36.3 (SD = 17.1). The mean total PIDA score was higher (43.9, SD = 16.1)) among subjects with self-perceived malocclusion than subjects without self-perceived malocclusion. Among the domains, the mean score for the PI domain was the highest (11.2, SD = 5.3) for all subjects, followed by the SI domain (11.1, SD = 6.9), the DSC domain (11.1, SD = 5.2), and the AC domain (2.9, SD = 1.8). A different trend of mean scores was observed in subjects with self-perceived malocclusion, with the reverse score of the DSC domain having the highest mean score (13.5, SD = 4.5), followed by the PI domain (13.4, SD = 5.1), SI (13.4, SD = 6.7), and AC domain (3.5, SD = 1.8). The subjects with self-perceived malocclusion had a significantly higher mean PIDA score than subjects without self-perceived malocclusion (*p* < 0.001).

### 3.4. Extent of Psychosocial Impact of Dental Aesthetics among the Participants by Self-Perceived Malocclusion

Overall, the extent of impacts (Table 4) was highest among those with one and two PIDA domains with significant impact (24.6% and 24.8%, respectively). 21.6% reported significant impacts on three domains, 16.8% on all domains, and 12.2% did not report significant impact on any domain. Amongst those with self-perceived malocclusion, the trend indicated that more subjects reported significant impacts in three and four domains (30.4% and 28.6%, respectively). Conversely, amongst those without self-perceived malocclusion, the majority tended to report significant impacts on only up to one or two domains (31.9% and 28.7%, respectively). Chi-square analysis showed that the differences in the extent of impacts between those with self-perceived malocclusion and those without self-perceived malocclusion was significant (*p* < 0.001).

### 3.5. Factors Associated with OHRQoL Related to Dental Aesthetics across Demographics of Malaysian Young Adults

In terms of the demographic factors associated with PIDA, Pearson correlation showed a weak positive correlation between age and PIDA scores (r = 0.038, *p* = 0.388). As for the other factors, Table 5 shows the results of multifactorial ANOVA of the significant factors associated with PIDA scores.

In the main effects model, gender, ethnic group and self-perceived malocclusion status were significantly associated with the PIDA scores (*p* < 0.05). Female subjects had a higher mean score compared to male subjects. Similarly, subjects with self-perceived malocclusion had a higher mean score compared to subjects without self-perceived malocclusion. Bonferroni post-hoc analysis on ethnic groups revealed that the mean PIDA scores of Malay and Indian subjects were significantly different (*p* = 0.002), with Malay subjects having the higher mean score. The effect size on gender and ethnic group factors were considered small (ηp2 < 0.06), while the effect size on self-perceived malocclusion status was considered large (ηp2 = 0.14). There was a significant interaction between gender and residence (*p* = 0.04) with a small effect size (ηp2 = 0.01). Further analysis of the adjusted mean with 95% confidence interval showed that the mean PIDA scores of subjects in urban areas were not significantly different, while the mean PIDA scores of females in sub-urban and rural areas were significantly higher than their male counterparts (Table 6).

## 4. Discussion

This study provided baseline data on the impact of dental aesthetics on the OHRQoL of Malaysian young adults. The prevalence, severity and extent of PIDA among the subjects were high and more prevalent in subjects with self-perceived malocclusion than subjects without self-perceived malocclusion. Females were more severely affected compared to males. There were racial differences in the PIDA scores. In this study, the Malay subjects had higher PIDA scores and lower OHRQoL related to dental aesthetics compared to Indian subjects. Females in suburban and rural areas had similar trends compared to their male counterparts.

In this study, there was a lack of representation from “older” young adults (those 24 years and older). This could be due to the difficulties in recruiting older students compared to the younger students. Most of the selected institutions have a limited number of postgraduate students who would have represented the older young adults. Furthermore, they may also have entered the workforce and have left or have not entered tertiary education institutes. Restrictions in obtaining demographic data from the institutions for confidentiality reasons had limited our ability to plan the sampling method. However, the issues did not affect data analysis since the presence of homogeneity in the variance and post-hoc test was done accordingly.

The reported overall prevalence of PIDA in this study was 87.8%. This was slightly lower than the prevalence of 90.2% which was reported in a group of adolescents in a previous study [10], suggesting that there was a reduction in impact with age. A past study showed that the impact in the domains of PIDA reduced with increasing age [20]. Nonetheless, the close gap between the two figures indicates that there was still a large proportion of Malaysian young adults who have negative impacts over malocclusion related OHRQoL. Furthermore, the order of prevalence by domain in this study was similar to that of the Malaysian adolescents [10], with PI domain having the highest prevalence, followed by DSC, SI and AC domains [10]. This suggests that Malaysian youths are generally most impacted within the domain that measures the negative emotions of dental aesthetics [12]. Subjects with self-perceived malocclusion notably also had higher prevalence as found in another study [9]. The trends were also reflected in the severity and extent of impacts.

In the current study, the overall mean PIDA score was 36.31 ± 17.07. A higher mean was noted in subjects with self-perceived malocclusion (43.90 ± 16.06). A similar trend was noticed in past studies [8,12,13], where the mean scores from groups with self-perceived need scored higher than the ones who did not. The PI domain had the highest mean, followed by SI, reverse-scored DSC and AC domains.

The extent of the impacts for all subjects was highest in two of the four PIDA domains (24.8%), and lowest in domains with no significant impact (12.2%), demonstrating the high extent of impacts among Malaysian young adults. Subjects with and without self-perceived malocclusion had significant impacts in three out of four domains (30.4%) and one out of four domains (31.9%) respectively. These were analogous to the findings amongst Malaysian adolescents [10].

The age of subjects was found to have no significant correlation with PIDA scores. This contradict the findings from a previous study where the psychosocial impacts reduced with age [21]. However, the age factor accounts for only 1.2% in predicting PIDA [21]. The contradictory results may be attributed to the fact that only young adults participated in this study, but the previous study involved adolescent and adult (12 to 39 years) participants [21], thus the young adults might not have much difference of impacts among their age group.

Meanwhile, income and residence factors were found to have no significant associations with PIDA scores in this study. This result may be related to the extensive subsidized medical and healthcare services with 95% of basic services available (dental clinics included) in Peninsular Malaysia and 70% in the Borneo region [22].

Factors that were significantly associated with PIDA in this study were gender, ethnic group and self-perceived malocclusion status. Women were found to have higher PIDA scores, in which similar findings had been shown in other studies [3,20]. This may be due to their higher awareness over their public image compared to men. As for ethnic group, total PIDA scores of Chinese subjects were not significantly different compared to PIDA scores of Malay and Indian subjects. However, Malay subjects were was found to have higher total PIDA scores than Indian subjects. Another study in Singapore [23] also found ethnic group as a factor, which found Indians to be more impacted by their malocclusion.

The current study also found significant interaction between gender and residence. In urban areas, males were found to have no significant differences in their OHRQoL compared to females, which suggests that they may have already assimilated with city life and embraced a metrosexual lifestyle, which corresponds to another study [24]. However, the males in sub-urban and rural areas were found to have lower impacts on their OHRQoL. Females in both areas were more impacted in their OHRQoL presumably since it is a common assumption that females are more concerned with appearance.

Based on the findings of the current study, clinicians, health policy makers and researchers will be able to enhance their understanding of the impacts of malocclusion towards the OHRQoL of young adults. In countries like Malaysia and the United Kingdom, government funded orthodontic treatment is limited for patients up to 18 years old and adults are considered for treatment on a case-by-case basis [25,26]. This has led to an escalation of fake braces provisions by predatory dental quacks to vulnerable young adults who cannot afford private treatment [25]. The trend of high oral impact from malocclusion that continues from adolescence [10] until young adult age as seen in this study reveals a continuation of negative influence of malocclusion on well-being into adulthood. Adults have reported improvement in the quality of life through orthodontic treatment [27]. Thus, health policy makers in countries that support age restriction for orthodontic services should acknowledge the impact of malocclusion in adults and consider the possibility of extending the provision of subsidized orthodontic treatment to young adults.

## 5. Conclusions

The prevalence, severity and extent of impact of OHRQoL on Malaysian young adults were high. Young adults with self-perceived malocclusion had significantly higher impacts than those without. Gender and ethnic group factors were significantly associated with total PIDA scores. Thus, the null hypotheses were rejected.

## Figures and Tables

**Table 1:** Socio-demographic characteristics of the subjects including presence of self-perceived malocclusion (*N* = 524).

Variables	Group	% (*N*)
Gender	Male	45.8 (240)
	Female	54.2 (284)
Age/year	18	27.9 (146)
	19	25.2 (132)
	20	30.0 (157)
	21	11.4 (60)
	22	3.4 (18)
	23	0.9 (5)
	24	0.4 (2)
	27	0.2 (1)
	30	0.6 (3)
Race	Malay	88.8 (465)
	Chinese	4.0 (21)
	Indian	5.7 (30)
	Others	1.5 (8)
Household Income (MYR)	≤499	7.3 (38)
	500–999	6.3 (33)
	1000–1499	15.1 (79)
	1500–1999	14.1 (74)
	2000–2499	12.6 (66)
	2500–2999	9.0 (47)
	3000–3499	10.9 (57)
	3500–3999	3.4 (18)
	4000–4999	5.1 (27)
	≥5000	16.2 (85)
Place of residence	Urban	57.1 (299)
	Sub-urban	23.3 (122)
	Rural	19.6 (103)
Self-perceived malocclusion	Yes	58.6 (217)
	No	41.4 (307)

Legend: MYR = Malaysia Ringgit.

**Table 2:** Summary on the prevalence of Psychosocial Impact of Dental Aesthetics (PIDA) in the sample (*N* = 524).

Variables	Overall *n* (%)	Self-Perceived Malocclusion (*n* = 217) *n* (%)	No Self-Perceived Malocclusion (*n* = 307) *n* (%)	*p*-Value ^a^
Total PIDA	460 (87.8)	201 (92.6)	259 (84.4)	0.004 *
Psychological impact	397 (75.8)	182 (83.9)	215 (70.0)	<0.0001 *
Dental self-confidence	311 (59.4)	170 (78.3)	141 (45.9)	<0.0001 *
Social impact	256 (48.9)	133 (61.3)	123 (40.1)	<0.0001 *
Aesthetic concern	116 (22.1)	76 (35.0)	40 (13.0)	<0.0001 *

Legend: ^a^ Chi-square test; * level of significance was set at *p* < 0.05.

**Table 3:** Distribution of scores for total PIDA and its domains amongst the study subjects and by self-perceived malocclusion status (*N* = 524).

Variables	All (*n* = 524)				Self-Perceived Malocclusion (*n* = 217)				No Self-Perceived Malocclusion (*n* = 307)				*p*-Value ^a^
	Mean	SD	Median	IQR	Mean	SD	Median	IQR	Mean	SD	Median	IQR	
Total PIDA	36.3	17.1	43.9	23	43.9	16.1	44.0	21	31.0	15.7	30.0	19	<0.001 *
PI	11.2	5.3	13.4	7	13.4	5.1	13.0	7	9.6	4.9	9.0	6	<0.001 *
DSC (R)	11.1	5.2	13.5	8	13.5	4.5	14.0	9	9.4	4.9	9.0	7	<0.001 *
SI	11.1	6.9	13.4	9	13.4	6.7	13.0	6	9.5	6.5	9.0	8	<0.001 *
AC	2.9	1.8	3.5	2	3.5	1.8	4.0	3	2.4	1.7	2.0	2	<0.001 *

Legend: ^a^ Independent t-test; * level of significance was set at *p* < 0.05; PIDA (Psychosocial impact of dental aesthetics); PI (Psychological impact); DSC (R) (Reverse score of Dental self-confidence); AC (Aesthetic concern).

**Table 4:** Extent of PIDA and its domains amongst the study subjects and by self-perceived malocclusion (*N* = 524).

Number of Domains with Significant Impact in ≥1 Item	Overall (*n* = 524) % (*n*)	Self-Perceived Malocclusion (*n* = 217) % (*n*)	No Self-Perceived Malocclusion (*n* = 307) % (*n*)	*p*-Value ^a^
0	12.2 (64)	7.4 (16)	15.6 (48)	<0.001 *
1	24.6 (129)	14.3 (31)	31.9 (98)	
2	24.8 (130)	19.4 (42)	28.7 (88)	
3	21.6 (113)	30.4 (66)	15.3 (47)	
4	16.8 (88)	28.6 (62)	8.5 (26)	

Legend: ^a^ Chi-square test; * level of significance was set at *p* < 0.05.

**Table 5:** Association between significant factors and total PIDA scores by multifactorial ANOVA analysis (*N* = 524).

Variables	Group	Adjusted Mean (95% CI)	F-Stat (df)	*p*-Value	Effect Size	Effect Size Descriptor
Gender	Male	30.45 (26.46,34.43)	18.18 (1)	<0.001 *	0.04	Small
	Female	35.31 (31.45,39.16)				
Race	Malay	37.39 (35.68,39.11)	4.27 (3)	0.005 *	0.03	Small
	Chinese	33.90 (27.10,40.71)				
	Indian	27.59 (21.77,33.41)				
	Others	32.62 (21.57,43.67)				
Household Income (MYR)	499 and below	32.19 (26.26,38.11)	0.52 (9)	0.858	0.01	Small
	500–999	35.05 (28.72,41.39)				
	1000–1499	35.19 (30.57,39.81)				
	1500–1999	34.69 (29.77,39.60)				
	2000–2499	33.71 (28.62,38.80)				
	2500–2999	33.45 (27.95,38.95)				
	3000–3499	31.29 (25.88,36.70)				
	3500–3999	29.31 (21.35,37.39)				
	4000–4999	30.50 (23.57,37.43)				
	5000 and above	33.34 (28.45,38.23)				
Place of residence	Urban	33.10 (29.32,36.88)	0.17 (2)	0.878	0.00	Small
	Sub-urban	32.30 (27.96,36.64)				
	Rural	33.23 (28.61,37.85)				
Self-perceived malocclusion status	Yes	36.16 (35.16,43.11)	81.26 (1)	<0.001 *	0.14	Large
	No	26.59 (22.76,30.43)				
Gender × Residence			3.18 (2)	0.043 *	0.01	Small

Legend: * *p* < 0.05; Gender × Residence as the significant interaction in the main model; MYR (Malaysian Ringgit).

**Table 6:** The adjusted mean PIDA scores for interaction of gender and residence.

Gender	Residence	Mean	95% Confidence Interval	
			Lower Bound	Upper Bound
Male	Urban	32.25	27.96	36.53
	Sub-urban	28.88 *	23.84	33.92
	Rural	27.08 *	20.92	33.24
Female	Urban	32.24	30.16	38.32
	Sub-urban	35.88 *	30.62	41.14
	Rural	37.90 *	32.81	43.00

Legend: * statistically significant.

## Data Availability

The data presented in this study are available on request from the corresponding author.
